# Association between Fruit and Vegetable Intakes and Mental Health in the Australian Diabetes Obesity and Lifestyle Cohort

**DOI:** 10.3390/nu13051447

**Published:** 2021-04-24

**Authors:** Joanna Rees, Simone Radavelli Bagatini, Johnny Lo, Jonathan M. Hodgson, Claus T. Christophersen, Robin M. Daly, Dianna J. Magliano, Jonathan E. Shaw, Marc Sim, Catherine P. Bondonno, Lauren C. Blekkenhorst, Joanne M. Dickson, Joshua R. Lewis, Amanda Devine

**Affiliations:** 1 Institute for Nutrition Research, Edith Cowan University, Perth 6027, Australia; s.radavellibagatini@ecu.edu.au (S.R.B.); jonathan.hodgson@ecu.edu.au (J.M.H.); marc.sim@ecu.edu.au (M.S.); c.bondonno@ecu.edu.au (C.P.B.); l.blekkenhorst@ecu.edu.au (L.C.B.); joshua.lewis@ecu.edu.au (J.R.L.); a.devine@ecu.edu.au (A.D.); 2 School of Medical and Health Sciences, Edith Cowan University, Perth 6027, Australia; c.christophersen@ecu.edu.au; 3 School of Science, Edith Cowan University, Perth 6027, Australia; j.lo@ecu.edu.au; 4 Medical School, University of Western Australia, Perth 6009, Australia; 5 Centre for Integrative Metabolomics & Computational Biology, School of Science, Edith Cowan University, Perth 6027, Australia; 6 WA Human Microbiome Collaboration Centre, School of Molecular & Life Sciences, Curtin University, Perth 6102, Australia; 7 Institute for Physical Activity and Nutrition, School of Exercise and Nutrition Science, Deakin University, Melbourne 3125, Australia; robin.daly@deakin.edu.au; 8 Clinical Diabetes and Epidemiology, Baker Heart and Diabetes Institute, Melbourne 3004, Australia; Dianna.Magliano@baker.edu.au (D.J.M.); Jonathan.Shaw@baker.edu.au (J.E.S.); 9 Psychology Discipline, School of Arts and Humanities, Edith Cowan University, Perth 6027, Australia; j.dickson@ecu.edu.au; 10 Centre for Kidney Research, Children’s Hospital at Westmead, School of Public Health, Sydney Medical School, The University of Sydney, Sydney 2145, Australia

**Keywords:** fruits, vegetables, dietary fibre, resistant starch, mental health

## Abstract

Increasing prevalence of mental health disorders within the Australian population is a serious public health issue. Adequate intake of fruits and vegetables (FV), dietary fibre (DF) and resistant starch (RS) is associated with better mental and physical health. Few longitudinal studies exist exploring the temporal relationship. Using a validated food frequency questionnaire, we examined baseline FV intakes of 5845 Australian adults from the AusDiab study and estimated food group-derived DF and RS using data from the literature. Perceived mental health was assessed at baseline and 5 year follow up using SF-36 mental component summary scores (MCS). We conducted baseline cross-sectional analysis and prospective analysis of baseline dietary intake with perceived mental health at 5 years. Higher baseline FV and FV-derived DF and RS intakes were associated with better 5 year MCS (*p* < 0.001). A higher FV intake (754 g/d vs. 251 g/d, Q4 vs. Q1) at baseline had 41% lower odds (OR = 0.59: 95% CI 0.46–0.75) of MCS below population average (<47) at 5 year follow up. Findings were similar for FV-derived DF and RS. An inverse association was observed with discretionary food-derived DF and RS. This demonstrates the association between higher intakes of FV and FV-derived DF and RS with better 5 year mental health outcomes. Further RCTs are necessary to understand mechanisms that underlie this association including elucidation of causal effects.

## 1. Introduction

The 2017–2018 Australian National Health Survey (AHS) reported that 20% of the Australian adult population have experienced a mental health disorder in the past 12 months, an increase of 17.5% since the 2014–2015 health survey [1]. It is essential to understand why the prevalence is rising and to identify modifiable risk factors that could direct future interventions to improve mental health and well-being [2]. There is a strong argument for studying mental health in the general population, as ‘symptom severity’ may represent a risk factor for the development of future clinical disorders over time, such as major depression [3]. Even moderate levels of anxiety and depression in non-clinical studies have been shown to interfere with normal functioning [3] and to impact negatively on social functioning [4], and interpersonal relationships [5]. As such, symptom severity in the general population is worthy of research attention.

Studies have attributed the growth of mental health disorders to increasing unhealthy lifestyles and Western diets that are highly processed, energy dense and low in dietary fibre (DF) and nutrient poor [6]. The 2017–2018 AHS reported that 95% of adults did not eat the recommended daily serves of fruit and vegetables (FV) [1]. For adults suffering from depression, the mental health benefits of consuming a healthy diet have been demonstrated by randomised controlled trials such as the SMILES trial [7] and HELFIMED [8], where diets rich in FV led to positive changes in mental health symptoms. A recent systematic review of 61 observational studies of adults from the general population reported that greater daily consumption of FV could be protective against depressive symptoms and promote higher levels of optimism and perceived social-emotional well-being [9]. The authors concluded that although a positive association was evident, more research is needed to further clarify the individual properties of FV that play a contributing role.

In addition to their healthy phytochemical and nutrient content, FV also provide a good source of DF. The advantages of consuming adequate DF to maintain digestive health and to reduce the risk of developing chronic disease have been well established [9,10,11,12]. Recently, the mechanisms underpinning these benefits have been researched in relation to the gut microbiota and their interaction with human metabolism [13]. It has been suggested that diets that are high in fermentable DF provide the substrate for microbial production of beneficial metabolites, short chain fatty acids (SCFA) [14]. SCFA have been reported to potentially influence gut-brain communication and brain function as well as modulating many host metabolic and immune responses [15,16,17,18]. Certain whole FV provide one of the best sources of this substrate as they are high in both fermentable and non-fermentable fibre as well as resistant starch (RS) [19]. RS is a form of starch that resists digestion by human enzymes providing an excellent source of fermentable DF that results in plentiful production of SCFA by the colonic gut microbiota [15,20]. Therefore, higher intakes of RS-rich foods support a favourable gut microbiota and increased SCFA production that is potentially associated with mental health benefits [15,21].

Poor diet quality, such as one high in processed foods and lacking in DF and FV, can contribute to an unbalanced gut microbiota that is known as dysbiosis [14,22]. Subsequently this can cause increased gut permeability, allowing for raised circulating inflammatory biomarkers and chronic low-grade inflammation that have been associated with the development of anxiety and depression [6,23,24,25]. While the beneficial relationship between FV, DF and positive mental health outcomes is widely reported in the literature, few studies have examined the components of FV, such as DF and RS, and their association with mental health symptoms over time [9,26,27,28].

The primary aim of this study was to investigate the relationship between daily consumption of FV and mental health symptoms 5 years later in a large cohort of Australian adults. Based on previous research, a secondary aim was to explore associations of DF and RS contributions from FV and other dietary components with mental health outcomes.

## 2. Subjects and Methods

### 2.1. Ethics Statement

The Human Research Ethics Committees of the International Diabetes Institute, Alfred Hospital and the Australian Institute of Health and Welfare approved the Australian Diabetes, Obesity and Lifestyle (AusDiab) study in accordance with the Helsinki Declaration of 1975 as revised in in 1983.

### 2.2. Study Population

The population included non-institutionalised Australian adults aged 25 years and above who participated in the AusDiab study. AusDiab was a national, population-based study examining the prevalence of diabetes, cardiovascular disease (CVD) and its risk factors, and kidney disease. Baseline measurements were collected from 1999 to 2000 on 11,247 individuals from 42 randomly selected urban and rural areas of Australia. Methods and response rates have been previously described [29]. In brief, 20,437 eligible participants completed a brief household interview and 11,247 subsequently attended a biomedical examination. Covariate data such as socio-economic index for areas (SEIFA), BMI, physical activity levels, smoking status, existing CVD, plasma diabetes status, marital status and education status were collected at either the household interview or the examination. This included an oral fasting glucose tolerance test, basic anthropometric tests and administration of questionnaires covering dietary intake and the 36-item short form survey (SF-36) health-related quality of life scale data. Of the 6400 participants who returned for the 5 year follow up (2004–2005), 5970 completed the SF-36 survey.

**Figure 1:**
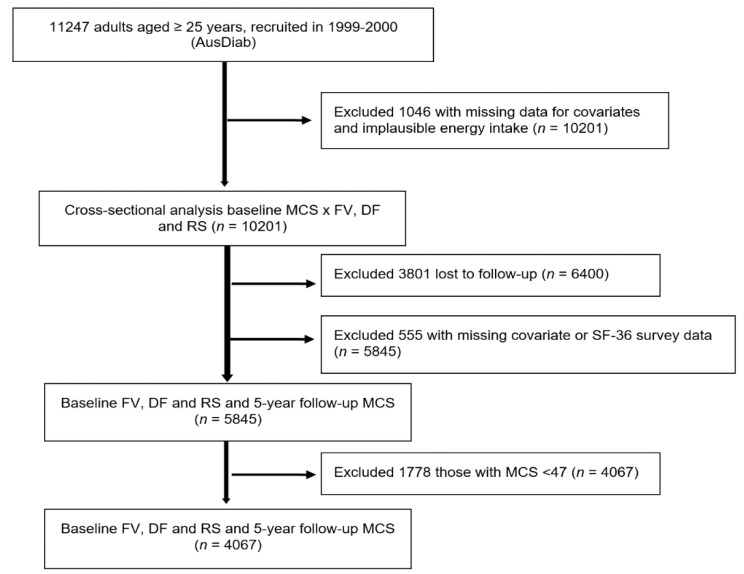
Study flow diagram to show exclusion steps involved and different analyses conducted. DF, dietary fibre; FV, fruit and vegetables combined; MCS, SF-36 Quality of Life Scale—Australian norm-based mental component score median; RS, resistant starch.

Individuals who had missing baseline data for any of the covariates and those who had extreme estimated total energy intake per day, either greater than 5000 kcal/day (20,900 kJ) or less than 500 kcal/day (2090 kJ), were excluded [30,31,32,33] (*n* = 1046), resulting in a sample of 10,201 for the cross-sectional analysis at baseline. For the longitudinal analysis, of the 6400 participants who returned for 5 year follow up, 555 either had missing covariate data or did not complete the SF-36 questionnaire and were excluded, leaving a prospective study sample size of 5845. For the analysis of the association between quartile intakes of FV intake at baseline and the likelihood of better mental health at 5 years, we further excluded those with baseline SF-36 mental component summary scores (MCS) below 47 (*n* = 1178) (Figure 1). The cut-off score of 47 was chosen as scores lower than 47 were interpreted to be below the average range for the general Australian population (explained in more detail below).

### 2.3. Dietary Assessment

Dietary intake was assessed at baseline (1999–2000) using a 74-item food frequency questionnaire (FFQ) developed by the Victorian Anti-Cancer Council of Australia for use in Australian adults [34,35,36,37]. Participants were asked to report their average intake of different food and beverage items over the previous 12 months, with 10 frequency response options ranging from “never” to “3 or more times per day”. Some foods such as cheese are further itemised into different types (hard cheese, soft cheese cottage cheese, etc.) and the FFQ output provides data for a total of 100 individual food and alcoholic beverage items [34,38]. These were categorised into food groups that included fruits, vegetables, discretionary foods, other foods and alcoholic beverages (Appendix A, Table A1). Total fruit included all fruits, fruit juice and tinned fruit (12 types). Total vegetables included all vegetables, potatoes, legumes, baked beans and other beans but excluded potatoes cooked in fat (hot chips) (23 types). Total discretionary foods included those which are not necessary for a healthy diet and are too high in saturated fat, added salt and/or added sugars, and low in DF [39] (21 types). Other foods included all the remaining foods captured by the FFQ (38 types). Alcoholic beverages (6 types) were included in total energy estimations but not in any other dietary intake analyses.

Total FV intake, separately and combined and DF and RS intake were estimated from the FFQ data. Output from the questionnaire provided data on daily intakes of each of the foods included in the questionnaire in grams (g) per day. The FFQ output provided data for overall total DF intake (g/day) calculated using the NUTTAB 95 nutrient data table for use in Australia [40]. However, these data were not used for our study as a more detailed calculation was necessary for the purposes of defining contributions from individual foods, rather than an overall total DF intake. This calculation used data from the literature [41] to identify intakes of specific DF types. The amount of insoluble fibre and soluble fibre for each food was estimated (g/day) and the two values summed to provide the total amount of DF for each food. Further, the amount of DF contributed by different food groups was estimated. These included DF from FV, DF from discretionary foods and DF from other foods (Appendix A, Table A1). As RS is not included in the data for insoluble or soluble fibre estimations, daily consumption of RS was classed as an independent entity and estimated separately, using an existing RS database that was created for and used in previous studies [20,42,43,44]. The amount of RS contributed by different food groups was estimated and included RS from FV, RS from discretionary foods and RS from other foods.

### 2.4. Outcome Variables—Mental Health and Well-Being

Mental health was measured at baseline (1999–2000) and 5 year follow up (2004–2005) using version 1 of the SF-36 [45] (https://www.rand.org/health-care/surveys_tools/mos/36-item-short-form.html (accessed on 23 April 2019)). The SF-36 is a generic outcome measure designed to examine a participant’s perceived health, well-being and mental health symptoms. It contains 36 items and is self-administered. All questions refer to the 4-week time period prior to completion of the survey. It encompasses eight domains of health-related quality of life: physical functioning; social functioning; role limitation due to physical functioning; bodily pain; general mental health; role limitation due to emotional functioning; vitality (energy and fatigue); and general health perception [45,46]. Responses from each domain provide relevant scores for that domain that are summed to produce raw scale scores ranging from 0–100, where 0 = worst health and 100 = best health. Scoring algorithms designed by the developers are applied to produce two summary component scores [45]. Of these, the MCS is the most valid to measure severity of mental health symptoms in the general population [47,48,49]. It incorporates responses from social functioning, general mental health and role limitations due to emotional functioning. For the SF-36, mental health is defined as the degree of nervousness or calmness and happiness or sadness felt [45,46,50,51]. The scoring algorithms used to create the MCS have been adapted for the general Australian population [52]. This results in norm-based MCS scores with a mean of 50 and standard deviation of 10, that can be compared to the distribution of scores for the general Australian population [52]. The SF-36 norm-based scoring algorithms suggest a group mean score below 47 to be the cut-off point, with scores lower than 47 to be interpreted as below the average range for the general Australian population, where lower scores represent increasing severity of mental health symptoms [47,50]. Therefore, for the statistical analysis of this study, where norm-based scoring was utilised, MCS scores were dichotomised at 47. Decreasing scores, from 46 down to the lowest possible score of zero, were deemed to represent increasing severity of mental health symptoms.

### 2.5. Statistical Analysis

Statistical analysis was performed using IBM SPSS Statistics for Windows, version 25.0 [53], Stata 15 [54] and R [55]. Descriptive statistics of normally distributed continuous variables were expressed as mean ± SD. Non-normally distributed continuous variables were expressed as median and interquartile range. Categorical variables were expressed as number and proportion (%). For the cross-sectional analysis logistic regression analysis using the survey command was used to examine the association between total FV intake and MCS at baseline. For the longitudinal analysis, the primary outcome of interest was follow-up MCS score (Australian norm-based) at 5 years. As the data were positively skewed, generalised linear models with a Gamma distribution and log-link were used to examine the association between baseline FV intake (g/d) and follow-up MCS. To investigate potential non-linearity of the relationship between the exposure and the outcome, logistic regression models were used with restricted cubic splines to investigate the relationship between quartiles of baseline FV intake (g/d) and binary measures of MCS (≥47 vs. <47) at 5 years of follow up; and the relationship between quartiles of baseline FV intake (g/d) and changes in MCS over 5 years. OR’s for follow-up MCS score (≥47 vs. <47) were based on logistic regression models using the “mgcv” package in R. The shape of the OR (95% CI) to FV intake relationship against MCS (≥47 vs. <47), was determined over a range of FV intake values using restricted cubic splines with the quartile 1 median used as the reference point. P-values for OR’s were obtained using Wald tests; tests for nonlinearity using a likelihood ratio test to compare nested models, with and without the nonlinear terms for the exposure, were applied. For visual simplicity, in all graphs presented, the *x*-axis was truncated at 3 SD above the mean. Two models were adopted; (1) unadjusted, and (2) multivariable-adjusted that included age (years), sex (male/female), BMI (kg/m^2^), energy intake (kJ/day), SEIFA, level of education (never to some high school, completed university or equivalent), relationship status (married, de facto, i.e., a relationship between two people who are not married but are living together on a domestic basis, separated, divorced, widowed, never married), level of physical activity (sedentary, insufficient, sufficient), smoking status (current smoker, ex-smoker, non-smoker), self-reported prevalence of CVD (yes/no), and diagnosis of diabetes based on plasma glucose levels (known diabetes mellitus (KDM), newly diagnosed diabetes mellitus (NDM), impaired fasting glucose (IFG), impaired glucose tolerance (IGT), normal glucose levels). This statistical analysis was repeated to test the association between baseline DF and RS intake and 5 year MCS (≥47 vs. <47) and again to test the association between DF and RS from each food group and 5 year MCS (≥47 vs. <47). A further binary logistic regression was conducted to look at the odds of dropping below 47 at follow up in those who had a MCS score ≥ 47 at baseline. For these tests, continuous FV intake variables (in g/d) were divided by SD. A final stratified analysis was carried out to explore any differences between sexes in consideration of the expectation that FV intakes may be greater overall in males than females. Statistical significance was set at a two-sided type one error rate of *p* < 0.05 for all tests.

## 3. Results

### 3.1. Baseline Characteristics

Baseline characteristics for all demographic and dietary intake data for the 5845 participants with MCS data at 5 year follow up are presented in Table 1. Participants ranged in age from 25 to 88 years, the mean age was 51 ± 12.7 years and 55% (*n* = 3209) were female. Almost half (47%) of the participants did not meet the current physical activity guidelines of 150 min per week and 31% were overweight and 21% obese. However, the participants were mostly healthy as only 12% were current smokers, 7% had a previous diagnosis of CVD and 25% had abnormal glucose tolerance (KDM/NDM/IFG/IGT). The median (IQR) MCS score at baseline was 52.0 (45.0–55.9) and 30% (*n* = 1778) of participants scored below the population average cut-off score of 47. Mean DF consumption of 17.2 (± 6.8) g/d for females and 20.7 (± 8.1) g/d for males, were both below the Australian government’s recommendation of 25 and 30 g per day for adult women and men, respectively [56]. FV and other foods both contributed 43% of total mean DF intake.

#### 3.1.1. Cross-Sectional Analysis at Baseline

Results from the cross-sectional analysis (*n* = 10,201) showed that higher baseline FV intakes, combined and separately, were positively associated with higher baseline MCS (*p* < 0.01 for all). There were similar associations for baseline intakes of DF from FV, total RS and RS from FV with baseline MCS (*p* = 0.03, *p* = 0.08 and *p* < 0.01, respectively) (Appendix B, Table A2).

#### 3.1.2. Longitudinal Analysis

**Figure 2:**
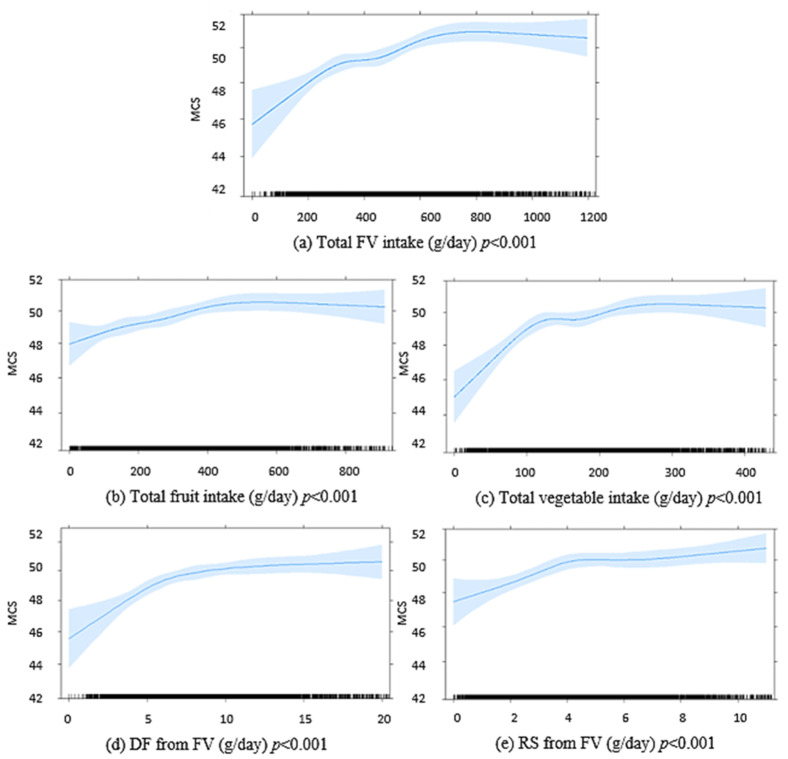
Graphic presentation from multivariable-adjusted general linear models of the longitudinal relationship between baseline intakes of (**a**) FV combined, (**b**) fruits, (**c**) vegetables, (**d**) DF from FV, (**e**) RS from FV, in g/day, with 5 year MCS scores in 5845 Australian adults from the AusDiab study. Models were adjusted for age, sex, BMI, energy intake, relationship status, physical activity, level of education, the socio-economic index for areas (SEIFA), diabetes and self-reported prevalence of CVD. The blue shaded areas represent 95% confidence intervals, the rug plot along the bottom of each graph represents each dietary intake observation. DF, dietary fibre; FV, fruit and vegetables combined; MCS, SF-36 Quality of Life Scale—Australian norm-based mental component score median; RS, resistant starch.

**Figure A1:**
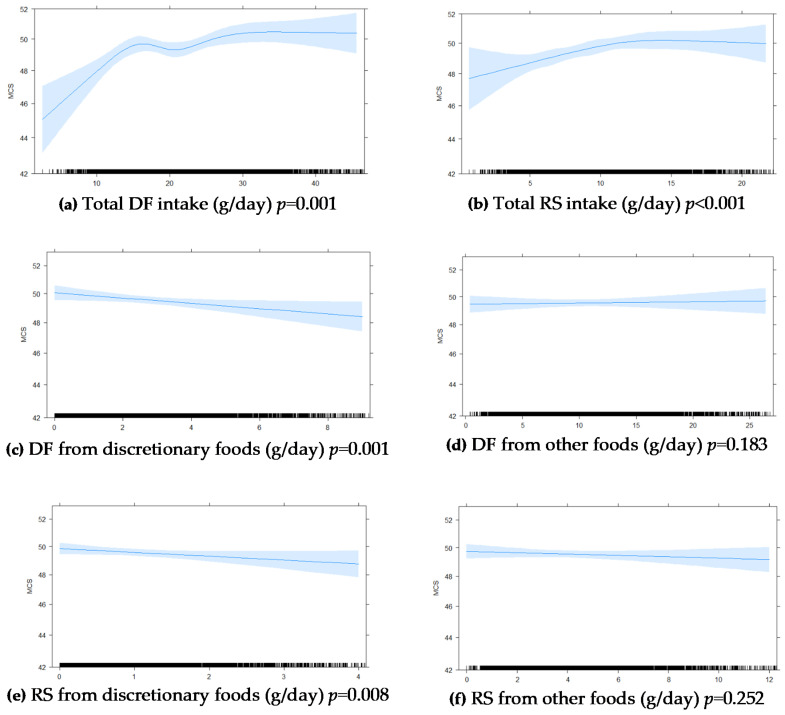
Graphic presentation from multivariable-adjusted general linear models of the longitudinal relationship between baseline intakes of (**a**) total DF, (**b**) total RS, (**c**) DF from discretionary foods, (**d**) DF from other foods, (**e**) RS from discretionary foods, (**f**) RS from other foods in (g/day), with 5 year MCS scores in 5845 Australian adults from the AusDiab study. Models were adjusted for age, sex, BMI, energy intake, relationship status, physical activity, level of education, the socio-economic index for areas (SEIFA), diabetes and self-reported prevalence of CVD. The blue shaded areas represent 95% CI, the rug plot along the bottom of each graph represents each dietary intake observation. DF, dietary fibre; FV, fruit and vegetables combined; MCS, SF-36 Quality of Life Scale—Australian norm-based mental component score median; RS, resistant starch.

A total of 5845 participants completed the SF-36 at 5 year follow up and were included in the analysis of the longitudinal relationship between baseline FV intake and 5-year MCS. A comparison between those included in the longitudinal analysis and those who did not complete 5-year SF 36 is shown in Supplemental Table S1. The median (IQR) MCS score at 5 years was 52.5 (45.3–56.2). In multivariable-adjusted linear regression, higher baseline FV intakes, separately and combined, were associated with higher MCS scores at 5 years (Figure 2). Higher FV-derived DF and FV-derived RS intakes at baseline were also positively associated with MCS scores at 5 years (Figure 2). An inverse association was seen for discretionary food-derived DF and RS intakes with MCS at 5 years. DF and RS from other foods were not significantly associated with MCS at 5 years (Appendix C, Figure A1).

**Figure 3:**
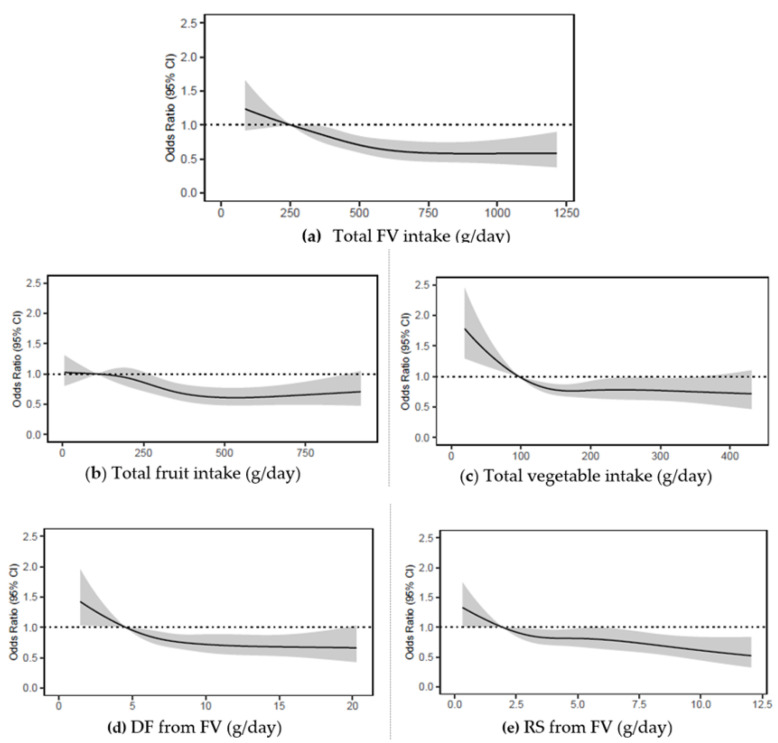
Odds ratios from logistic regression models with restricted cubic spline curves describing the association of baseline intakes of (**a**) FV combined, (**b**) fruits, (**c**) vegetables, (**d**) DF from FV, (**e**) RS from FV in g/day, with 5-year MCS scores (vertical axis) in 4067 Australian adults from the AusDiab study (excludes those with baseline MCS score < 47). Odds ratios are based on models adjusting for age, sex, BMI, energy intake, relationship status, physical activity, level of education, the socio-economic index for areas (SEIFA), smoking status, diabetes status, and self-reported history of CVD, and are comparing the specific level of intake (horizontal axis) to the median (IQR) intake for participants in the lowest intake quartile (FV combined: 251 g/day; fruits: 106 g/day; vegetables: 97 g/day); DF from FV: 5 g/day; RS from FV: 2 g/day. DF, dietary fibre; FV, fruit and vegetables combined; MCS, SF-36 Quality of Life Scale—Australian norm-based mental component score median; RS, resistant starch.

**Figure A2:**
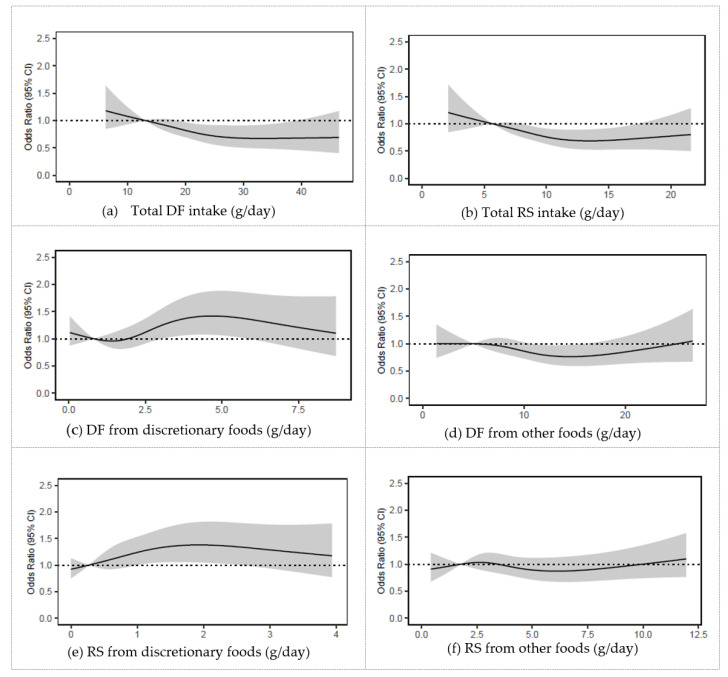
Odds ratios from logistic regression models with restricted cubic spline curves describing the association between baseline consumption of (**a**) total DF, (**b**) total RS, (**c**) DF from discretionary foods, (**d**) DF from other foods, (**e**) RS discretionary foods, (**f**) RS from other foods, in g/d with 5 year MCS scores (vertical axis) in 4067 Australian adults from the AusDiab study (excludes those with baseline MCS score < 47). Odds ratios are based on models adjusting for age, sex, BMI, energy intake, relationship status, physical activity, level of education, the socio-economic index for areas (SEIFA), smoking status, diabetes status, and self-reported history of CVD, and are comparing the specific level of intake (horizontal axis) to the median (IQR) intake for participants in the lowest intake quartile of total DF: 13 g/day; total RS: 6 g/day; DF from discretionary foods: 0.8 g/day); DF from other foods: 5 g/day; RS from discretionary foods 0.3 g/day; RS from other foods: 1.8 g/day. DF, dietary fibre; MCS, SF 36 Quality of Life Scale mental component summary scores (Australian norm-based); RS, resistant starch.

After excluding the 1178 participants who had baseline MCS scores below 47, higher FV intake was associated with greater likelihood of having better mental health at 5 years (Figure 3, Table 2 and Supplemental Table S2). Similar findings were observed for fruit and vegetable intakes separately (Figure 3, Table 2 and Supplemental Table S2) and for total DF and total RS intakes (Appendix D, Figure A2 and Table A3)**.** Food group analysis showed that higher intakes of FV-derived DF and FV-derived RS were associated with greater likelihood of having better mental health at 5 years (Figure 3 and Table 2). In contrast, higher intakes of DF and RS from discretionary foods were associated with greater likelihood of having below population average mental health at 5 years (Appendix D, Figure A2 and Table A3). There was no association between DF and RS from other foods and 5-year MCS scores. We also found that when comparing MCS scores at baseline with corresponding scores at follow up, a positive change in score was significantly associated with baseline intakes of FV, DF and DF from FV, and RS and RS from FV (all *p* < 0.01) (Supplemental Table S3).

#### 3.1.3. Sensitivity Analysis

After excluding the 1178 participants who had baseline MCS scores below 47, sensitivity analyses of 1912 males and 2155 females showed that FV intakes were not significantly different between sexes (*p* = 0.200). There were significant differences in between-sex intakes of discretionary food-derived RS (*p* = 0.035) and other food-derived DF and RS (*p* = 0.002, *p* < 0.001, respectively). For males, higher intakes of DF and RS from discretionary foods were associated with greater likelihood of having below population average mental health at 5 years (*p* < 0.001, *p* = 0.037, respectively) whereas for females, there was no significant association between intakes and 5-year MCS score (Supplemental Table S4).

## 4. Discussion

In this large study of Australian men and women across the adult lifespan, we found that higher intakes of FV at baseline (1999–2000) were associated with significantly healthier SF-36 MCS scores at baseline and at 5 year follow up, even after adjusting for lifestyle factors such as physical activity levels, CVD history, diabetes status and smoking. At baseline, an increase of approximately one serve of vegetables per day was associated with a significant reduction in the likelihood of dropping below the population average for mental health at 5 years. This aligns with results from a recent study of FV intake in the UK [57] that found a dose–response improvement in mental health and well-being with increasing quantity and frequency of FV intake. The benefits of FV intake were emphasised further by our food group analysis, in particular results from the analysis of FV-derived DF and RS. Those who consumed greater FV-derived DF and RS were more likely to have an MCS score equal to or above the population average 5 years later. A positive association was observed between higher intakes of both FV-derived DF and FV-derived RS and MCS scores at 5 year follow up. These findings highlight the importance of a diet rich in FV and FV-derived DF and RS, for maintaining good mental health.

Where there is increasing evidence to support the positive outcomes for those with a healthy diet pattern, the detrimental effect of poor diet quality has been associated with negative consequences for mental health [2,58]. In a 2020 review of DF and depressive symptoms, inflammation was suggested to be a potential mediator between DF and mental health symptoms [6]. The review summarised that a high-quality diet, such as one high in DF and notably FV, was linked to lower circulating inflammatory markers which reduced the risk of depression [28]. In contrast to the positive associations found between FV-derived DF and RS, the participants in our study who had higher intakes of DF and RS from discretionary foods were less likely to have MCS scores equal to or above the population average. An inverse association was observed between higher discretionary food-derived DF and RS, and perceived mental health status at 5 year follow up. These findings were confirmed by Akbaraly et al. (2009) [59], who expanded the focus from single nutrients to whole foods and found that diets of higher quality which incorporated frequent FV intake were correlated with less depressive symptoms over 5 years [59].

Interestingly, when comparing differences in intakes between sexes, our results were further emphasised for discretionary food intakes of males. Although FV intakes and therefore FV-derived DF intakes, were not significantly different between sexes, male participants in this cohort had higher intakes of DF and RS from discretionary foods than their female counterparts. For males, this higher discretionary food contribution to diet was associated with greater odds of having mental health below the population average at 5 year follow up that was not observed for females.

The interaction between FV-derived and/or discretionary food-derived DF and mental health can possibly be explained by the gut-brain axis, which is the bidirectional relationship between the human gut microbiome and the brain [22,24,25,60,61,62]. Dietary intake is critical to the composition and function of the gut microbiome. Whole food sources rich in DF as well as other nutrients and phytochemicals, is necessary to support the beneficial microbes and their metabolites [62]. A healthy well-balanced gut microbiome is protective against the risk of developing a mental health disorder, whereas gut dysbiosis has been linked to raised inflammation and the presence of stress, anxiety and depression [2,17,19]. Our results provide further insight into the concept that regular and adequate FV intake can contribute to improved mental health outcomes [9]. Given that a high-quality diet can impact the diversity and composition of the gut microflora and thereby reduce detrimental circulating inflammatory mediators, a diet rich in FV can lead to positive outcomes for both gut microbiome and mental health.

The results observed for RS intakes offer further support for the benefits of FV. RS found predominantly in fruits, vegetables and grains is known to facilitate increased microbial production of beneficial metabolites such as short chain fatty acids that are associated with physical and mental health benefits [20,25]. In our study, those in the highest quartile for intakes of FV-derived RS at baseline, were more likely to have an MCS score equal to or above the population average 5 years later, than those who were in the lowest quartile. Although there was no significant association observed between DF or RS from other foods with MCS at follow up, the importance of foods such as grains and cereals cannot be overlooked, particularly those that are high in DF and RS. The relationship between grains and cereals with mental health was outside the remit for this study. However, these foods provide a valuable source of DF that are essential for digestive health and have been proven to reduce the risk of developing chronic disease and morbidity [63]. Our findings of a positive association between follow-up mental health and total DF and RS intakes suggest there is a benefit from greater consumption, but studies involving more in-depth dietary analysis are needed [44].

### Strengths and Limitations

There are several strengths to our study. Firstly, this study comprised a large population-based, national cohort of male and female Australian adults across a broad age range, with mental health measured at two timepoints (i.e., at baseline and after 5 years). Secondly, the findings provide valuable information surrounding the beneficial relationship between greater consumption of FV and DF and perceived mental health, that are reinforced by positive findings from the analysis of food group specific DF and RS intakes. Lastly, our results endorse the benefit of adequate FV consumption for short-term and longer-term mental health outcomes and emphasise the importance of diet quality, i.e., one that is high in FV and low in discretionary foods, for mental health within the adult population.

The authors recognise that the association between diet and mental health faces causal challenges [64] and are affected by a multitude of contributing factors that may not have been captured here. We took measures to exclude those with greater severity of mental health symptoms at baseline, thereby reducing the potential reverse causality bias. Secondly, the data utilised in this study may have introduced self-report bias. However, as the data were used in conjunction with the other data collected, this is reduced [65]. Thirdly, the analyses of FV, DF and RS were limited due to the simplistic nature of the FFQ. Ideally, additional descriptive information on the types of foods, how they are cooked and how they are eaten is required to gain a more comprehensive understanding of the relationship [44]. Many inconsistencies exist between available RS datasets and the foods they contain, and therefore further studies should be conducted once more accurate and up-to-date data are released [20].

## 5. Conclusions

It is becoming increasingly recognised that nutrition is a critical component for consideration when addressing mental health and well-being. Our study demonstrates the improved outcomes for 5 year mental health that are associated with higher intakes of FV and FV-derived DF and RS. These results advocate for future interventions to investigate the causal effects of healthier dietary behaviours for improving mental health outcomes. In particular, the significant positive association of FV-derived DF and RS and the inverse association with discretionary foods, highlight the importance of further research to explore the influence of dietary patterns and quality on mechanisms that drive mental health. Continued efforts should be made to encourage increased FV consumption at a population level to assist with modifying the risk factors of developing mental health issues over time.

## Figures and Tables

**Table 1:** Baseline characteristics of covariables and dietary intakes of 5845 Australian adults from the AusDiab study.

Age (years)	51.4 ± 12.7
Sex	
Male	2636 (45)
Female	3209 (55)
SEIFA	1021.3 ± 86.0
BMI (kg/m^2^)	26.9 ± 4.8
BMI category	
Underweight (>18.5 kg/m^2^)	42 (0.7)
Healthy weight (18.5–24.9 kg/m^2^)	2748 (47)
Overweight (25.0–29.9 kg/m^2^)	1805 (31)
Obese (≥30 kg/m^2^)	1250 (21)
Relationship status	
Married	4459 (76)
De facto ^	241 (4)
Separated	126 (2)
Divorced	327 (6)
Widowed	289 (5)
Never married	403 (7)
Level of education	
Never to some high school	2173 (37)
University or equivalent	3726 (63)
Level of physical activity	
Sedentary (0 min/week)	916 (16)
Insufficient (<150 min/week)	1812 (31)
Sufficient (≥150 min/week)	3117 (53)
Smoking status	
Current smoker	670 (12)
Ex-smoker	1738 (30)
Never smoked	3437 (59)
Diabetes	
Known diabetes mellitus	209 (4)
Newly diagnosed diabetes mellitus	204 (4)
Impaired fasting glucose	339 (6)
Impaired glucose tolerance	687 (12)
Normal glucose levels	4406 (75)
Prevalent CVD	393 (7)
SF-36 MCS score	49.3 ± 9.5
DIETARY INTAKE	
^1^ Total energy intake (kJ/day)	8475.6 ± 3019.5
Total FV intake (g/day)	500.3 ± 241.1
Total fruit intake (g/day)	302.7 ± 203.7
Total vegetable intake (g/day)	197.5 ± 89.8
Total DF intake (g/day)	18.8 ± 7.6
Total RS intake (g/day)	9.5 ± 4.0
Total DF from FV (g/day)	7.9 ± 3.7
Total DF from discretionary foods (g/day)	1.9 ± 1.5
Total DF from other food (g/day)	8.5 ± 5.1
Total RS from FV (g/day)	4.4 ± 2.5
Total RS from discretionary foods (g/day)	1.1 ± 1.0
Total RS from other food (g/day)	3.9 ± 2.7

^ de facto, a relationship between two people who are not married but are living together on a domestic basis. ^1^ Energy intake includes energy from alcoholic beverages. Values are percentages, *n* (%), the means ± SDs. CVD, cardiovascular disease; DF, dietary fibre; FV, fruit and vegetables combined; MCS, SF-36 mental component summary scores; RS, resistant starch; SEIFA, socio-economic index for areas; BMI, body mass index.

**Table 2:** Association of baseline intakes of FV, fruit, vegetable, DF from FV and RS from FV, with 5-year MCS scores in 4 067 Australian adults from the AudDiab study (excludes those with baseline MCS score < 47).

Median Quartiles	OR	95% CI
Combined FV intake (g/d)		
Q1 (251.3)	Reference	
Q2 (391.0)	0.83	0.71–0.96
Q3 (523.9)	0.68	0.57–0.82
Q4 (754.0)	0.59	0.46–0.75
Fruit intake (g/d)		
Q1 (106.0)	Reference	
Q2 (219.5)	0.91	0.76–1.08
Q3 (338.6)	0.72	0.59–0.86
Q4 (532.2)	0.61	0.48–0.77
Vegetable intake (g/d)		
Q1 (96.8)	Reference	
Q2 (149.0)	0.78	0.69–0.88
Q3 (197.6)	0.77	0.65–0.92
Q4 (274.3)	0.78	0.61–0.98
DF from FV (g/d)		
Q1 (4.55)	Reference	
Q2 (6.87)	0.81	0.71–0.93
Q3 (9.10)	0.74	0.61–0.88
Q4 (12.98)	0.69	0.54–0.88
RS from FV (g/d)		
Q1 (1.9)	Reference	
Q2 (3.4)	0.84	0.72–0.97
Q3 (4.9)	0.81	0.68–0.98
Q4 (7.4)	0.74	0.59–0.93

Estimated using logistic regression, Q1 is reference, multivariable adjusted for age, sex, BMI, energy intake, relationship status, physical activity, level of education, the socio-economic index for areas (SEIFA), diabetes and self-reported prevalence of CVD. DF, dietary fibre; FV, fruit and vegetables combined; MCS, SF-36 Quality of Life Scale—Australian norm-based mental component score median; RS, resistant starch.

## Data Availability

Data described in the manuscript will be made available upon reasonable request upon application and approval of authors.

## Supplementary Materials

The following are available online at https://www.mdpi.com/article/10.3390/nu13051447/s1, Table S1: Comparison between baseline characteristics of covariables and dietary intakes of 5 845 Australian adults from the AusDiab study who completed SF-36 at 5-year follow-up and 4 356 who did not Table S2. Association of standard deviation differences in baseline dietary intakes with 5 year MCS score < 47 by in 4067 Australian adults from the AusDiab study (excludes those with MCS score <47 at baseline). Table S3: Change in MCS scores from baseline to 5 year follow up by quartile dietary intakes at baseline in 4067 Australian adults from the AusDiab study (excludes those with MCS score <47 at baseline). Table S4: Association of baseline intakes of total DF, total RS, DF from FV, RS from FV, DF from discretionary foods, DF from other foods, DF from discretionary foods and RS from other foods, with 5 year MCS scores in 2155 female and 1912 male Australian adults from the AusDiab study (excludes those with baseline MCS score <47).

## Appendix A

**Table A1:** Food frequency questionnaire food groups.

Other Foods	Vegetables	Fruit	Discretionary Foods
Allbran/Bran flakes	Avocado	Oranges	Butter
Weetbix	Potatoes	Apples	Butter and margarine blends
Cornflakes	Tomatoes	Pears	Tomato sauce
Porridge	Capsicum	Bananas	Jam
Muesli	Lettuce	Melon	Vegemite
Rice	Cucumber	Pineapple	Sugar
Pasta	Celery	Strawberries	Sweet biscuits
Crackers	Beetroot	Apricots	Cakes
White bread	Carrots	Peaches	Meat pies
Wholemeal bread	Cabbage	Mango	Pizza
Rye bread	Cauliflower	Tinned fruit	Hamburger
Multigrain bread	Broccoli	Fruit juice	Ham
High-fibre white bread	Spinach		Salami
Margarine	Peas		Sausages
Polyunsaturated margarine	Green beans		Bacon
Monounsaturated margarine	Bean sprouts		Fried fish
Hard cheese	Baked beans		Chocolate
Firm cheese	Other beans		Flavoured milk drink
Soft cheese	Pumpkin		Ice cream
Ricotta or cottage cheese	Onion		Hot chips
Cream cheese	Garlic		Crisps
Low-fat cheese	Mushrooms		
Full-cream milk	Zucchini		
Reduced-fat milk			
Skim milk			
Yoghurt			
Eggs			
Nuts			
Peanut butter			
Soya milk			
Tofu			
Tinned fish			
Fish			
Beef			
Veal			
Chicken			
Lamb			
Pork			

## Appendix B

**Table A2:** The cross-sectional association of dietary intakes with MCS scores at baseline in 10,201 Australian adults from the AusDiab study.

*n* = 10,201	Mean (SD)	^1^*p* Value
Total FV (g/d)	500.3 ± 241.1	0.003
Total fruit (g/d)	302.7 ± 203.7	<0.001
Total vegetables (g/d)	197.5 ± 89.8	<0.001
Total DF (g/d)	18.8 ± 7.6	0.338
DF from FV (g/d)	7.9 ± 3.7	0.030
DF from discretionary foods (g/d)	1.9 ± 1.5	0.120
DF from other foods (g/d)	18.8 ± 7.6	0.384
Total RS (g/d)	9.5 ± 4.0	0.080
RS from FV (g/d)	4.4 ± 2.5	0.005
RS from discretionary foods (g/d)	1.2 ± 1.0	0.052
RS from other foods (g/d)	3.8 ± 2.7	0.147

^1^*p* values are for Q4 with Q1 as reference. Values are the mean ± SDs. Logistic regression analysis adjusted for age, sex, BMI, energy intake, relationship status, physical activity, level of education, the socio-economic index for areas (SEIFA), diabetes and self-reported prevalence of CVD. DF, dietary fibre; FV, fruit and vegetables combined; MCS, SF-36 Quality of Life Scale—Australian norm-based mental component score median; RS, resistant starch.

## Appendix D

**Table A3:** Association of baseline intakes of total DF and RS, discretionary food-derived DF and RS and other food-derived DF and RS with 5 year MCS scores in 4067 Australian adults from the AusDiab study (excludes those with baseline MCS score < 47).

Median Quartiles	OR	95% CI
Total DF (g/d)		
Q1 (13.1)	Reference	
Q2 (18.4)	0.86	0.74–1.00
Q3 (23.0)	0.75	0.61–0.93
Q4 (30.9)	0.67	0.50–0.91
Total RS (g/d)		
Q1 (5.7)	Reference	
Q2 (8.1)	0.86	0.75–0.99
Q3 (10.4)	0.74	0.61–0.90
Q4 (14.2)	0.69	0.53–0.90
DF discretionary foods (g/d)		
Q1 (0.8)	Reference	
Q2 (1.7)	0.97	0.81–1.16
Q3 (2.7)	1.18	0.96–1.45
Q4 (4.9)	1.42	1.07–1.88
DF from other foods (g/d)		
Q1 (5.0)	Reference	
Q2 (8.1)	0.94	0.81–1.10
Q3 (11.3)	0.81	0.67–0.99
Q4 (16.5)	0.78	0.60–1.01
RS discretionary foods (g/d)		
Q1 (0.3)	Reference	
Q2 (0.6)	1.11	0.92–1.33
Q3 (1.1)	1.26	1.02–1.56
Q4 (2.1)	1.38	1.04–1.82
RS from other foods (g/d)		
Q1 (1.8)	Reference	
Q2 (2.9)	1.03	0.87–1.21
Q3 (4.3)	0.93	0.77–1.13
Q4 (6.7)	0.88	0.67–1.14

Estimated using logistic regression, Q1 is reference, adjusted for age, sex, BMI, energy intake, relationship status, physical activity, level of education, the socio-economic index for areas (SEIFA), diabetes and self-reported prevalence of CVD. DF, dietary fibre; MCS, SF-36 Quality of Life Scale—Australian norm-based mental component score median; RS, resistant starch.
