# Supplementary material for: Association between Fruit and Vegetable Intakes and Mental Health in the Australian Diabetes Obesity and Lifestyle Cohort
Source: Nutrients. 2021 Apr 24;13(5):1447. doi: 10.3390/nu13051447 (PMC8146262; doi:10.3390/nu13051447)
Supplement: Supplementary file 1 [file nutrients-13-01447-s001.zip › nutrients-1163873-supplementary.pdf]

Association between fruit and vegetable intakes and mental health in the Australian Diabetes Obesity and Lifestyle (AusDiab) cohort

Corresponding author: Joanna Rees,

Address: Edith Cowan University, 270 Joondalup Drive, Joondalup, Western Australia 6027.

Telephone: +61 403 872 227

Email: j.rees@ecu.edu.au

Online supplemental material

Supplemental Table S1. Comparison between baseline characteristics of covariables and dietary intakes of 5 845 Australian adults from the AusDiab study who completed SF-36 at 5-year follow-up and 4 356 who did not.

	**Without MCS at 5-year follow-up**	**With MCS at 5-year follow-up**
Age (years)	51.5 ± 16.2	51.4 ± 12.7
Sex		
Male	1944 (45)	2636 (45)
Female	2412 (55)	3209 (55)
SEIFA	1004.0 ± 93.2	1021.3 ± 86.0
BMI (kg/m^2)	27.1 ± 5.1	26.9 ± 4.8
BMI category		
Underweight (>18.5 kg/m^2)	52 (1.2)	42 (0.7)
Healthy weight (18.5 – 24.9 kg/m^2)	1965 (45)	2748 (47)
Overweight (25.0 – 29.9 kg/m^2)	1338 (31)	1805 (31)
Obese (≥ 30 kg/m^2)	1001 (23)	1250 (21)
Relationship status		
Married	2877 (66)	4459 (76)
De facto^	241 (6)	241 (4)
Separated	137 (3)	126 (2)
Divorced	291 (7)	327 (6)
Widowed	374 (9)	289 (5)
Never married	436 (10)	403 (7)
Level of education		
Never to some high school	1975 (45)	2173 (37)
University or equivalent	2381 (55)	3726 (63)
Level of physical activity		
Sedentary (0 min/week)	829 (19)	916 (16)
Insufficient (< 150 min/week)	1325 (30)	1812 (31)
Sufficient (≥150 min/week)	2202 (51)	3117 (53)
Smoking status		
Current smoker	923 (21)	670 (12)
Ex-smoker	1250 (29)	1738 (30)
Never smoked	2183 (50)	3437 (59)
Diabetes		
Known diabetes mellitus	214 (5)	209 (4)
Newly diagnosed diabetes mellitus	250 (6)	204 (4)
Impaired fasting glucose	577 (13)	339 (6)
Impaired glucose tolerance	218 (5)	687 (12)
Normal glucose levels	3097 (71)	4406 (75)
Prevalent CVD	446 (10)	393 (7)
SF-36 MCS score	47.9 ± 10.4	49.3 ± 9.5
DIETARY INTAKE		
[1]Total energy intake (kJ/day)	8387.6 ± 3233.1	8475.6 ± 3019.5
Total FV intake (g/day)	460.2 ± 241.1	481.4 ± 238.8
Total fruit intake (g/day)	290.6 ± 205.5	302.7 ± 203.7
Total vegetable intake (g/day)	169.6 ± 84.5	178.7 ± 83.7
Total DF from FV (g/day)	7.9 ± 3.9	7.9 ± 3.7
Total RS from FV (g/day)	4.2 ± 2.5	4.4 ± 2.5

^ de facto, a relationship between two people who are not married but are living together on a domestic basis; [1]Energy intake includes energy from alcoholic beverages. Values are percentages, *n* (%), means ± SD's. CVD, cardiovascular disease; DF, dietary fibre; FV, fruit and vegetables combined; MCS, SF-36 mental component summary score; RS, resistant starch; SEIFA, socio-economic index for areas

Supplemental Table S2. Association of standard deviation differences in baseline dietary intakes with 5 year MCS scores < 47 by in 4 067 Australian adults from the AusDiab study (excludes those with MCS scores < 47 at baseline).

Dietary intake (g/d)	OR	[1]*p* value	95% CI
Total FV (g/d)	0.82	<0.001	0.74 - 0.91
Total fruit (g/d)	0.86	0.002	0.78 – 0.94
Total vegetables (g/d)s	0.88	0.005	0.80 – 0.96
Total DF (g/d)	0.87	0.032	0.77 – 0.99
DF from FV (g/d)	0.86	0.003	0.79 – 0.95
DF from fruit (g/d)	0.91	0.052	0.83 – 1.00
DF from vegetables (g/d)	0.88	0.007	0.80 – 0.99
DF from discretionary foods (g/d)	1.08	0.192	0.96 – 1.22
DF from other foods (g/d)	0.96	0.409	0.86 – 1.06
Total RS (g/d)	0.89	0.043	0.80 – 1.00
RS from FV (g/d)	0.86	0.001	0.78 – 0.94
RS from fruit (g/d)	0.90	0.016	0.82 – 0.98
RS from vegetables (g/d)	0.90	0.025	0.82 – 0.99
RS from discretionary foods (g/d)	1.05	0.371	0.95 – 1.16
RS from other foods (g/d)	1.02	0.771	0.92 – 1.12
Total discretionary foods (g/d)	1.15	0.046	1.00 – 1.31

[1]*p* values are for Q4 with Q1 as reference. Estimated using logistic regression Q1 is reference, multivariable adjusted for age, sex, BMI, energy intake, relationship status, physical activity, level of education, SEIFA (socio-economical index for areas), diabetes and self-reported prevalence of CVD. DF, dietary fibre; FV, fruit and vegetables combined; MCS, SF-36 Quality of Life Scale – Australian norm based mental component score median; RS, resistant starch.

Supplemental Table S3. Change in MCS scores from baseline to 5 year follow up by quartile dietary intakes at baseline in 4 067 Australian adults from the AusDiab study (excludes those with MCS scores < 47 at baseline).

Dietary intake (g/d)	[1]*p* value	95% Confidence Interval
Total FV (g/d)	0.004	0.31 – 1.66
Total fruit (g/d)	0.021	0.11 – 1.44
Total vegetables (g/d)	0.003	0.33 – 1.65
Total DF (g/d)	0.004	0.37 – 2.02
DF from FV (g/d)	0.001	0.44 – 1.78
DF from fruit (g/d)	0.400	-0.37 – 0.94
DF from vegetables (g/d)	<0.000	0.53 – 1.84
DF from discretionary foods (g/d)	0.897	-0.84 – 0.74
DF from other foods (g/d)	0.547	-0.51 – 0.96
Total RS (g/d)	0.069	-0.05 – 1.48
RS from FV (g/d)	<0.000	0.58 – 1.88
RS from fruit (g/d)	0.114	-0.12 – 1.14
RS from vegetables (g/d)	0.001	0.49 – 1.83
RS from discretionary foods (g/d)	0.434	-0.46 – 1.06
RS from other foods (g/d)	0.834	-0.66 – 0.81

[1]*p* values are for Q4 with Q1 as reference. Binary logistic regression adjusted for age, sex, BMI, energy intake, relationship status, physical activity, level of education, SEIFA (socio-economical index for areas), diabetes and self-reported prevalence of CVD. DF, dietary fibre; FV, fruit and vegetables combined; MCS, SF-36 Quality of Life Scale – Australian norm based mental component score median; RS, resistant starch.

Supplemental Table S4. Association of baseline intakes of total DF, total RS, DF from FV, RS from FV, DF from discretionary foods, DF from other foods, DF from discretionary foods and RS from other foods, with 5 year MCS scores in 2 155 female and 1 912 male Australian adults from the AusDiab study (excludes those with baseline MCS scores < 47).

Male (n = 1912)			Female (n = 2155)		
**Median quartiles**	**OR**	**95% CI**	**Median quartiles**	**OR**	**95% CI**
Total DF (g/d)			Total DF (g/d)		
Q1 (14.6)	Reference		Q1 (12.3)	Reference	
Q2 (20.5)	0.88	0.71 – 1.10	Q2 (16.9)	0.79	0.65 – 0.96
Q3 (25.7)	0.71	0.52 – 0.98	Q3 (21.1)	0.77	0.58 – 1.02
Q4 (34.6)	0.62	0.62 – 0.96	Q4 (27.4)	0.78	0.53 – 1.15
Total RS (g/d)			Total RS (g/d)		
Q1 (6.5)	Reference		Q1 (5.1)	Reference	
Q2 (9.1)	0.77	0.64 – 0.93	Q2 (7.4)	0.92	0.75 – 1.14
Q3 (11.6)	0.74	0.55 – 0.98	Q3 (9.5)	0.76	0.58 – 1.00
Q4 (15.5)	0.72	0.49 – 1.06	Q4(12.9)	0.70	0.48 – 1.01
DF from FV (g/d)			DF from FV (g/d)		
Q1 (4.6)	Reference		Q1 (4.5)	Reference	
Q2 (7.0)	0.94	0.75 – 1.17	Q2 (6.8)	0.71	0.60 – 0.86
Q3 (9.5)	0.86	0.65 – 1.13	Q3 (8.9)	0.63	0.50 – 0.81
Q4 (13.9)	0.78	0.55 – 1.12	Q4 (12.2)	0.60	0.43 – 0.84
RS from FV (g/d)			RS from FV (g/d)		
Q1 (2.1)	Reference		Q1 (1.9)	Reference	
Q2 (3.6)	0.88	0.71 – 1.09	Q2 (3.3)	0.79	0.65 – 0.96
Q3 (5.1)	0.86	0.66 – 1.13	Q3 (4.7)	0.78	0.61 – 1.01
Q4 (7.8)	0.84	0.59 – 1.17	Q4 (7.0)	0.66	0.48 – 0.92
DF from discretionary foods (g/d)			DF from discretionary foods (g/d)		

Q1 (1.1)	Reference		Q1 (0.7)	Reference	
Q2 (2.3)	1.07	0.80 – 1.42	Q2 (1.4)	0.99	0.77 – 1.26
Q3 (3.6)	1.49	1.08 – 2.06	Q3 (2.1)	1.02	0.77 – 1.35
Q4 (6.0)	1.76	1.16 – 2.66	Q4 (3.9)	1.07	0.74 – 1.56
DF from other foods (g/d)			DF from other foods (g/d)		
Q1 (5.6)	Reference		Q1 (4.7)	Reference	
Q2 (9.4)	0.76	0.62 – 0.95	Q2 (7.3)	1.06	0.86 – 1.31
Q3 (12.7)	0.68	0.52 – 0.90	Q3 (10.0)	0.95	0.73 – 1.24
Q4 (18.7)	0.67	0.46 – 0.98	Q4 (14.3)	0.98	0.69 – 1.40
RS discretionary foods (g/d)			RS discretionary foods (g/d)		
Q1 (0.4)	Reference		Q1 (0.2)	Reference	
Q2 (0.8)	1.33	1.00 – 1.76	Q2 (0.5)	0.99	0.78 – 1.28
Q3 (1.4)	1.52	1.10 – 2.09	Q3 (0.8)	1.07	0.80 – 1.42
Q4 (2.5)	1.54	1.03 – 2.29	Q4 (1.5)	1.17	0.81 – 1.67
RS other foods (g/d)			RS other foods (g/d)		
Q1 (2.0)	Reference		Q1 (1.6)	Reference	
Q2 (3.4)	0.79	0.63 – 1.00	Q2 (2.6)	1.21	0.95 – 1.54
Q3 (4.9)	0.68	0.51 – 0.91	Q3 (3.8)	1.24	0.94 – 1.65
Q4 (7.6)	0.64	0.64 – 0.93	Q4 (5.8)	1.31	0.91 – 1.89

Estimated using logistic regression, Q1 is reference, adjusted for age, sex, BMI, energy intake, relationship status, physical activity, level of education, SEIFA (socio-economical index for areas), diabetes and self-reported prevalence of CVD. DF, dietary fibre; FV, fruit and vegetables combined; MCS, SF-36 Quality of Life Scale – Australian norm based mental component score median; RS, resistant starch
